# Di-4-pyridylmethane­diol

**DOI:** 10.1107/S1600536808018588

**Published:** 2008-06-28

**Authors:** Warren R. Knapp, Robert L. LaDuca

**Affiliations:** a Lyman Briggs College, Department of Chemistry, Michigan State University, East Lansing, MI 48825, USA

## Abstract

In the title compound, C_11_H_10_N_2_O_2_, individual mol­ecules lie across crystallographic twofold rotation axes. Neighboring mol­ecules engage in O—H⋯N hydrogen bonding, forming square-grid layers parallel to the *ab* plane.

## Related literature

For related literature, see: Chen & Mak (2005 ▶); Montney *et al.* (2008 ▶); Zaworotko (2007 ▶).
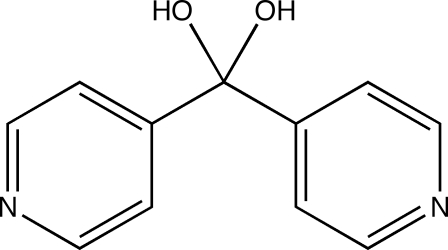


## Experimental

### 

#### Crystal data

C_11_H_10_N_2_O_2_
*M* *_r_* = 202.21
Tetragonal, 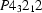

*a* = 7.6130 (2) Å
*c* = 17.5864 (11) Å
*V* = 1019.27 (7) Å^3^
*Z* = 4
Mo *K*α radiation
μ = 0.09 mm^−1^
*T* = 173 (2) K
0.30 × 0.22 × 0.16 mm

#### Data collection

Bruker APEXII diffractometer
Absorption correction: multi-scan (*SADABS*; Sheldrick, 1996 ▶) *T*_min_ = 0.686, *T*_max_ = 0.745 (expected range = 0.907–0.985)
14287 measured reflections
605 independent reflections
549 reflections with *I* > 2σ(*I*)
*R*_int_ = 0.040

#### Refinement

*R*[*F* ^2^ > 2σ(*F* ^2^)] = 0.029
*wR*(*F* ^2^) = 0.076
*S* = 1.13
605 reflections
72 parameters
H atoms treated by a mixture of independent and constrained refinement
Δρ_max_ = 0.13 e Å^−3^
Δρ_min_ = −0.13 e Å^−3^

### 

Data collection: *APEX2* (Bruker, 2006 ▶) and *COSMO* (Bruker, 2006 ▶); cell refinement: *APEX2*; data reduction: *SAINT* (Bruker, 2006 ▶); program(s) used to solve structure: *SHELXS97* (Sheldrick, 2008 ▶); program(s) used to refine structure: *SHELXL97* (Sheldrick, 2008 ▶); molecular graphics: *Crystal Maker* (Palmer, 2007 ▶); software used to prepare material for publication: *SHELXL97*.

## Supplementary Material

Crystal structure: contains datablocks I, global. DOI: 10.1107/S1600536808018588/hg2417sup1.cif

Structure factors: contains datablocks I. DOI: 10.1107/S1600536808018588/hg2417Isup2.hkl

Additional supplementary materials:  crystallographic information; 3D view; checkCIF report

## Figures and Tables

**Table 1:** Hydrogen-bond geometry (Å, °)

*D*—H⋯*A*	*D*—H	H⋯*A*	*D*⋯*A*	*D*—H⋯*A*
O1—H1*A*⋯N1^i^	0.87 (2)	1.87 (2)	2.7376 (19)	173.4 (19)

Symmetry code: (i) 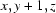.

**Fig. 1.**
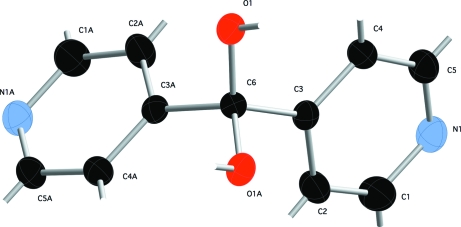
A complete molecule of the title compound. H atom positions are shown as gray sticks. Color code: C black, N blue, O red.

**Fig. 2.**
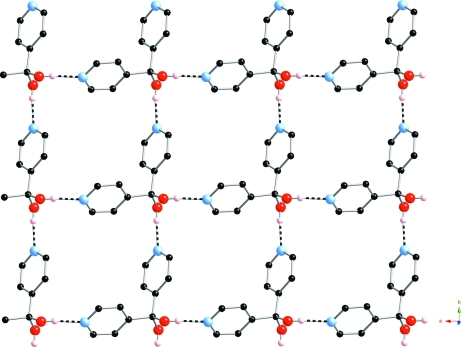
A view down c showing the aggregation of molecules of the title compound into a (4,4) square grid. Hydrogen bonding is indicated as dashed lines.

**Fig. 3.**
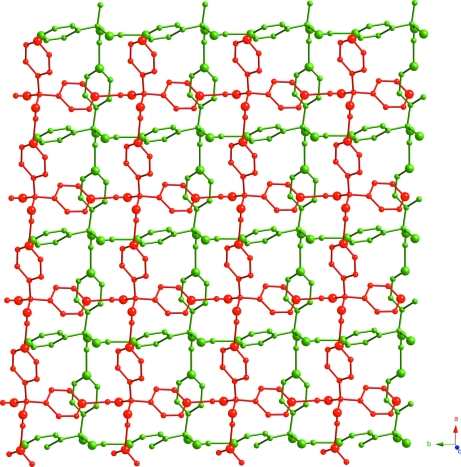
A view down c of the offset double layer motif in the title compound.

## supplementary crystallographic information

### Comment

While coordination polymers constructed from 4,4'-bipyridine are very common (Zaworotko, 2007), related phases based on its hydrogen-bonding capable analog di-4-pyridylketone (dpk) have not yet been reported (Montney *et al.*, 2008). In an attempt to prepare a zinc nitrate coordination polymer incorporating dpk through an aqueous solution method, an *in situ* hydration reaction took place, resulting in the crystallization of di(4-pyridyl)methanediol (dpmd).

Crystals of (I) crystallize in an noncentrosymmetric tetragonal space group, with an asymmetric unit consisting of one half of a dpmd molecule. Its central *sp*^3^ hybridized C atom rests on a crystallographic 2-fold rotation axis. Operation of this symmetry element generates a complete dpmd molecule (Figure 1).

Each molecule of (I) is conjoined to four others, two *via* O—H···N hydrogen bonding donation from its alcohol functional groups and two *via* O—H···N hydrogen bonding acceptance at its pyridyl N atoms. As a result, a grid-like layer motif is formed, which is parallel to the *ab* crystal plane (Figure 2).

Adjacent layer patterns aggregate through weak C—H···O interactions to construct double layer slab motifs (Figure 3). In turn the double slabs stack along the *c* crystal direction by packing forces to form the pseudo three-dimensional crystal structure of (I).

### Experimental

Zinc nitrate hexahydrate was obtained commercially. Di-4-pyridylketone (dpk) was prepared *via* a published procedure (Chen & Mak, 2005). Zinc nitrate hexahydrate (55 mg, 0.19 mmol) was dissolved in 3.0 ml water in a glass test tube. A 2 ml aliquot of a 1:1 water:methanol mixture was then added, followed by 3 ml of a methanolic solution of dpk (70 mg, 0.38 mmol). Colourless blocks of (I) were deposited after standing at 298 K for one week.

### Crystal data

**Table:**

C_11_H_10_N_2_O_2_	*Z* = 4
*M**_r_* = 202.21	*F*_000_ = 424
Tetragonal, *P*4_3_2_1_2	*D*_x_ = 1.318 Mg m^−^^3^
Hall symbol: P 4nw 2abw	Mo *K*α radiation λ = 0.71073 Å
*a* = 7.6130 (2) Å	Cell parameters from 14287 reflections
*b* = 7.6130 (2) Å	θ = 2.9–25.3º
*c* = 17.5864 (11) Å	µ = 0.09 mm^−^^1^
α = 90º	*T* = 173 (2) K
β = 90º	Block, colourless
γ = 90º	0.30 × 0.22 × 0.16 mm
*V* = 1019.27 (7) Å^3^	

### Data collection

**Table:**

Bruker APEXII diffractometer	605 independent reflections
Radiation source: fine-focus sealed tube	549 reflections with *I* > 2σ(*I*)
Monochromator: graphite	*R*_int_ = 0.040
*T* = 173(2) K	θ_max_ = 25.3º
ω and ψ scans	θ_min_ = 2.9º
Absorption correction: multi-scan(SADABS; Sheldrick, 1996)	*h* = −8→9
*T*_min_ = 0.686, *T*_max_ = 0.745	*k* = −9→9
14287 measured reflections	*l* = −21→20

### Refinement

**Table:**

Refinement on *F*^2^	Secondary atom site location: difference Fourier map
Least-squares matrix: full	Hydrogen site location: inferred from neighbouring sites
*R*[*F*^2^ > 2σ(*F*^2^)] = 0.029	H atoms treated by a mixture of independent and constrained refinement
*wR*(*F*^2^) = 0.076	*w* = 1/[σ^2^(*F*_o_^2^) + (0.0412*P*)^2^ + 0.1593*P*] where *P* = (*F*_o_^2^ + 2*F*_c_^2^)/3
*S* = 1.13	(Δ/σ)_max_ < 0.001
605 reflections	Δρ_max_ = 0.13 e Å^−^^3^
72 parameters	Δρ_min_ = −0.12 e Å^−^^3^
Primary atom site location: structure-invariant direct methods	Extinction correction: none

### Special details

**Table:**

Geometry. All e.s.d.'s (except the e.s.d. in the dihedral angle between two l.s. planes) are estimated using the full covariance matrix. The cell e.s.d.'s are taken into account individually in the estimation of e.s.d.'s in distances, angles and torsion angles; correlations between e.s.d.'s in cell parameters are only used when they are defined by crystal symmetry. An approximate (isotropic) treatment of cell e.s.d.'s is used for estimating e.s.d.'s involving l.s. planes.
Refinement. Refinement of *F*^2^ against ALL reflections. The weighted *R*-factor *wR* and goodness of fit *S* are based on *F*^2^, conventional *R*-factors *R* are based on *F*, with *F* set to zero for negative *F*^2^. The threshold expression of *F*^2^ > σ(*F*^2^) is used only for calculating *R*-factors(gt) *etc*. and is not relevant to the choice of reflections for refinement. *R*-factors based on *F*^2^ are statistically about twice as large as those based on *F*, and *R*- factors based on ALL data will be even larger.

### Fractional atomic coordinates and isotropic or equivalent isotropic displacement parameters (Å^2^)

**Table:**

	*x*	*y*	*z*	*U*_iso_*/*U*_eq_	
O1	0.62268 (16)	0.65611 (15)	0.06536 (7)	0.0256 (3)	
H1A	0.616 (3)	0.768 (3)	0.0580 (11)	0.031*	
N1	0.6238 (2)	0.01378 (19)	0.04930 (8)	0.0317 (4)	
C1	0.5269 (3)	0.1201 (3)	0.09305 (11)	0.0377 (5)	
H1	0.4681	0.0717	0.1344	0.045*	
C2	0.5099 (3)	0.2974 (2)	0.07980 (10)	0.0322 (5)	
H2	0.4422	0.3665	0.1121	0.039*	
C3	0.5945 (2)	0.3724 (2)	0.01785 (9)	0.0216 (4)	
C4	0.6967 (2)	0.2639 (2)	−0.02701 (10)	0.0263 (4)	
H4	0.7573	0.3089	−0.0686	0.032*	
C5	0.7075 (2)	0.0872 (2)	−0.00905 (11)	0.0307 (5)	
H5	0.7772	0.0157	−0.0395	0.037*	
C6	0.5671 (2)	0.5671 (2)	0.0000	0.0207 (5)	

### Atomic displacement parameters (Å^2^)

**Table:**

	*U*^11^	*U*^22^	*U*^33^	*U*^12^	*U*^13^	*U*^23^
O1	0.0303 (7)	0.0177 (6)	0.0287 (7)	−0.0006 (5)	−0.0056 (6)	−0.0012 (5)
N1	0.0390 (10)	0.0217 (8)	0.0343 (8)	0.0022 (7)	−0.0040 (8)	0.0004 (7)
C1	0.0455 (13)	0.0300 (11)	0.0377 (10)	0.0023 (10)	0.0098 (9)	0.0074 (9)
C2	0.0352 (11)	0.0266 (10)	0.0349 (10)	0.0056 (8)	0.0095 (9)	0.0023 (8)
C3	0.0196 (9)	0.0221 (9)	0.0232 (8)	−0.0007 (7)	−0.0047 (7)	−0.0016 (7)
C4	0.0267 (10)	0.0256 (10)	0.0264 (9)	0.0007 (8)	0.0018 (8)	−0.0017 (8)
C5	0.0342 (10)	0.0254 (10)	0.0325 (10)	0.0059 (8)	−0.0021 (9)	−0.0057 (9)
C6	0.0208 (8)	0.0208 (8)	0.0205 (11)	0.0008 (10)	0.0002 (7)	−0.0002 (7)

### Geometric parameters (Å, °)

**Table:**

O1—C6	1.3998 (17)	C3—C4	1.382 (2)
O1—H1A	0.87 (2)	C3—C6	1.529 (2)
N1—C5	1.331 (2)	C4—C5	1.384 (2)
N1—C1	1.338 (2)	C4—H4	0.9300
C1—C2	1.376 (3)	C5—H5	0.9300
C1—H1	0.9300	C6—O1^i^	1.3998 (17)
C2—C3	1.389 (2)	C6—C3^i^	1.529 (2)
C2—H2	0.9300		
			
C6—O1—H1A	109.7 (13)	C3—C4—H4	120.5
C5—N1—C1	116.94 (15)	C5—C4—H4	120.5
N1—C1—C2	123.20 (17)	N1—C5—C4	123.77 (17)
N1—C1—H1	118.4	N1—C5—H5	118.1
C2—C1—H1	118.4	C4—C5—H5	118.1
C1—C2—C3	119.53 (17)	O1—C6—O1^i^	112.43 (19)
C1—C2—H2	120.2	O1—C6—C3	105.03 (8)
C3—C2—H2	120.2	O1^i^—C6—C3	113.29 (8)
C4—C3—C2	117.59 (16)	O1—C6—C3^i^	113.30 (8)
C4—C3—C6	122.61 (14)	O1^i^—C6—C3^i^	105.03 (8)
C2—C3—C6	119.76 (14)	C3—C6—C3^i^	107.87 (19)
C3—C4—C5	118.94 (17)		
			
C5—N1—C1—C2	0.6 (3)	C3—C4—C5—N1	0.3 (3)
N1—C1—C2—C3	0.8 (3)	C4—C3—C6—O1	−123.43 (16)
C1—C2—C3—C4	−1.6 (3)	C2—C3—C6—O1	58.9 (2)
C1—C2—C3—C6	176.25 (17)	C4—C3—C6—O1^i^	−0.4 (2)
C2—C3—C4—C5	1.0 (3)	C2—C3—C6—O1^i^	−178.06 (15)
C6—C3—C4—C5	−176.72 (15)	C4—C3—C6—C3^i^	115.45 (17)
C1—N1—C5—C4	−1.1 (3)	C2—C3—C6—C3^i^	−62.25 (14)

Symmetry codes: (i) *y*, *x*, −*z*.

### Hydrogen-bond geometry (Å, °)

**Table:**

*D*—H···*A*	*D*—H	H···*A*	*D*···*A*	*D*—H···*A*
O1—H1A···N1^ii^	0.87 (2)	1.87 (2)	2.7376 (19)	173.4 (19)

Symmetry codes: (ii) *x*, *y*+1, *z*.
